# Cervical Cancer Screening with HPV Testing: Updates on the Recommendation

**DOI:** 10.1055/s-0041-1739314

**Published:** 2022-02-15

**Authors:** Carla Fabrine Carvalho, Julio Cesar Teixeira, Joana Froes Bragança, Sophie Derchain, Luiz Carlos Zeferino, Diama Bhadra Vale

**Affiliations:** 1 Department of Obstetrics and Gynecology, Universidade Estadual de Campinas, Campinas, SP, Brazil

**Keywords:** uterine cervical neoplasms, human papillomavirus DNA tests, early detection of cancer, accessibility of health services, mass screening, neoplasias do colo do útero, testes de DNA para papilomavírus humano, detecção precoce de câncer, acessibilidade dos serviços de saúde, rastreamento em massa

## Abstract

The present update is a reassessment of the 2018 ‘Guidelines for HPV-DNA Testing for Cervical Cancer Screening in Brazil’ (Zeferino et al.) 9 , according to the changes observed in new international guidelines and knowledge updates. The most relevant and recent guidelines were assessed. Questions regarding the clinical practice were formulated, and the answers considered the perspective of the public and private sectors of the Brazilian health system. The review addressed risk-based strategies regarding age to start and stop screening, the use of cytology and colposcopy to support management decisions, treatment, follow-up strategies, and screening in specific groups, including vaccinated women. The update aims to improve the prevention of cervical cancer and to reduce overtreatment and the misuse of HPV testing.

## Resumo

Esta atualização é uma reavaliação das “Recomendações para o uso de testes de DNA-HPV no rastreamento do câncer do colo do útero no Brasil” (Zeferino et al., 2018), 9 de acordo com as mudanças observadas nas novas recomendações internacionais, além das atualizações no conhecimento. As recomendações mais relevantes e recentes foram avaliadas. Questões referentes à prática clínica foram formuladas, e as respostas consideraram a perspectiva do sistema de saúde brasileiro, tanto público quanto privado. Esta revisão abrange estratégias baseadas em risco sobre idade para início e término de rastreamento, o uso da citologia e colposcopia para apoiar as condutas, tratamento, estratégias de seguimento, e rastreamento em grupos específicos, incluindo mulheres vacinadas. Esta atualização tem o objetivo de melhorar as estratégias de prevenção do câncer do colo de útero e reduzir o supertratamento e o uso incorreto dos testes de HPV.

## Introduction

Although cervical cancer is the fourth most common cancer among women worldwide, mortality rates are decreasing, mainly in high-income countries. 1 Improvements in screening, diagnosis and treatment are probably the reason for this decline. The rates of cervical cancer vary substantially among the different regions of Brazil due to the differences in access to health care. 2 3

According to the World Health Organization (WHO), eliminating cervical cancer depends on multiple efforts, including prevention through vaccination, screening and treatment of precursor lesions, and treatment and palliative care for invasive cervical cancer. 4 5 There is a consensus that cervical cancer screening is more effective when based on human papillomavirus (HPV) DNA tests over longer intervals. 6 7

In 2016, Instituto Nacional do Câncer (INCA, the Brazilian National Cancer Institute) updated its guidelines for cervical cancer screening. 8 Although HPV testing is not available at the national free-of-charge screening program, substantial misuse of the HPV-DNA test was happening at private clinics at that time. Concerned about that situation, a group of experts published in 2018 a recommendation for HPV testing in screening in Brazil. 9

There is a relevant discussion among clinicians if the guidelines should consider the management based on risk instead of primarily on test-based algorithms. The risk-based approach means that the probability of finding a case of cervical intraepithelial neoplasia 3 or more severe lesion (CIN 3 + ) would guide the practice, not solely the combination of test results.

The present review aims to reassess the 2018 recommendation for HPV-DNA testing in cervical cancer screening in Brazil, according to the changes observed in the new international guidelines and updates on HPV screening knowledge. The review considered the social circumstances of health care access in the country. Topics not addressed in the first recommendation are now discussed due to the increase in the use of HPV testing.

## Methods

For the present critical review, relevant guidelines were identified through a search on the PubMed (MEDLINE), CENTRAL (Cochrane), and Embase databases; the reference sections of the retrieved publications; and the websites of relevant organizations launched during the past five years. The following guidelines were accessed:

‘National Cervical Screening Program: Guidelines for the Management of Screen-Detected Abnormalities, Screening in Specific Populations, and Investigation of Abnormal Vaginal Bleeding’ (Cancer Council Australia, 2020); 10 ‘2019 ASCCP Risk-Based Management Consensus Guidelines for Abnormal Cervical Cancer Screening Tests and Cancer Precursors’ (Perkins et al., 2020); 11 ‘Cervical cancer screening and prevention’ (ACOG, 2016); 12 ‘Cervical cancer screening for individuals at average risk: 2020 guideline update from the American Cancer Society’ (Fontham et al., 2020); 13 ‘Cervical screening: ESGO-EFC position paper of the European Society of Gynaecologic Oncology (ESGO) and the European Federation of Colposcopy (EFC)’ (Kyrgiou et al., 2020); 14 ‘Diretrizes brasileiras para o rastreamento do câncer do colo do útero’ (INCA, 2016); 8 ; ‘Guidelines for HPV-DNA Testing for Cervical Cancer Screening in Brazil’ (Zeferino et al., 2018); 9 and ‘Screening for Cervical Cancer: US Preventive Services Task Force Recommendation Statement’ (Curry et al., 2018). 15

The guidelines were accessed to answer questions regarding the clinical practice from the Brazilian perspective. The reference sections of the publications and the medical literature databases were used to develop the answers. Access to care and relevant cultural practices were considered from the perspective of the Brazilian health system framework.

In the present review, we have considered HPV tests positive (HPV + ) when the woman gets a positive result for oncogenic types. The acronyms CYTO+ meant abnormal cytology and COLPO+ meant abnormal colposcopy. The question mark ('?') meant the topic regarded.

## Results

**Fig. 1:**
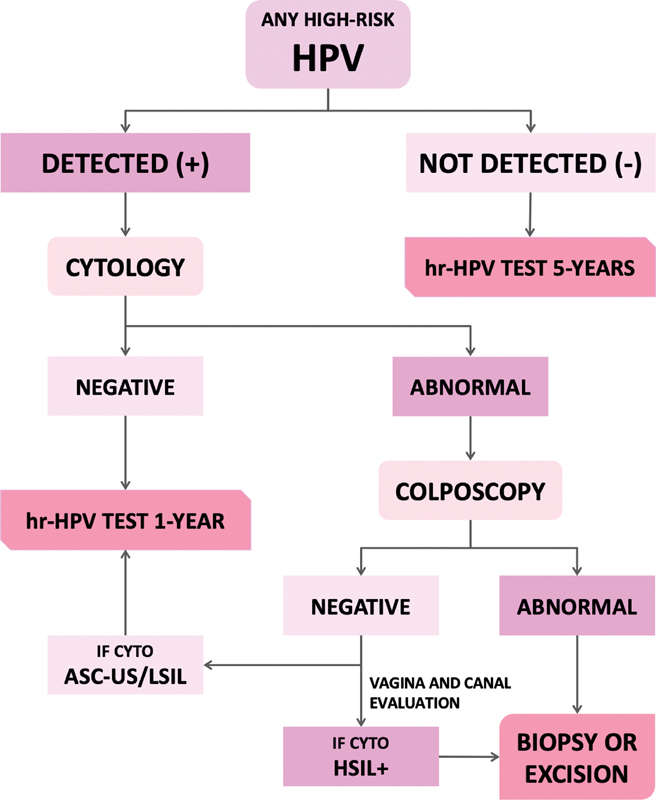
Suggestions regarding the management of HPV-based screening in women older than 25 years of age, when genotyping is not available or if types other than 16 or18 are detected. Abbreviations: Hr-HPV, high-risk HPV test; cyto, cytology; HSIL + , atypical squamous cells, cannot exclude a high-grade lesion (ASC-H); high-grade squamous intraepithelial lesion (HSIL); atypical glandular cells (AGCs); or adenocarcinoma in situ (AIS).

**Fig. 2:**
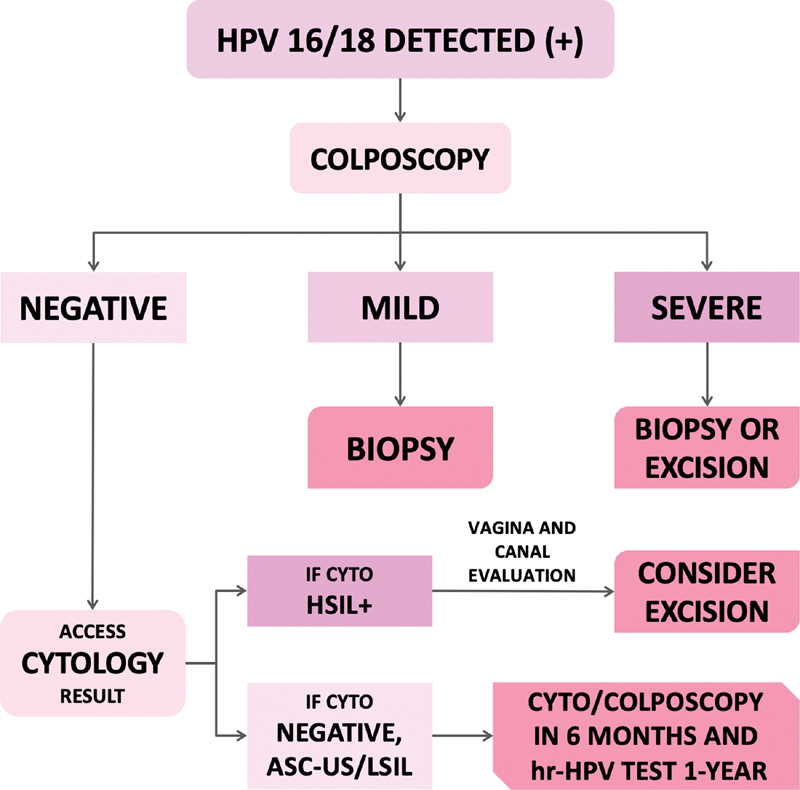
Suggestions regarding the management of HPV-based screening in women older than 25 years of age, when HPV 16 or 18 is detected. Hr-HPV: high-risk HPV test; cyto: cytology; HSIL + , atypical squamous cells, cannot exclude a high-grade lesion (ASC-H); high-grade squamous intraepithelial lesion (HSIL); atypical glandular cells (AGCs); or adenocarcinoma in situ (AIS).

A summary of the recommendations is presented in Figures 1 and 2 .

1. **Should HPV testing alone replace cytology for women older than 30 years?**

The WHO recommends screening based on HPV testing instead of cytology, when resources are available, since 2014. 16 For primary screening, the improvement in sensitivity reduces the rate of false-negative results. Human papillomavirus testing is recommended due to its higher sensitivity compared to cytology in the first screening; it detects more than 60% to 70% of cases of invasive cervical carcinoma compared to cytology-based screening. 6 Cytology alone is acceptable if there is no access to primary HPV testing. 9 13 16

Testing for HPV is more likely to detect adenocarcinoma precursor lesions than cytology-based screening. 6 17 18 With it, there is an increase in the proportion of adenocarcinomas detected, achieving a more efficient screening. 18 19

In high-income countries, HPV testing is cost-effective because of its higher negative predictive value combined with extended testing intervals. 20 21 22 23 In low and middle-income countries, cost-effectiveness must be addressed. The success of the screening programs in those countries is affected by factors not usually considered in high-income ones: the access of women to screening and further assessment and the lack of control in testing intervals. A population-based study 24 pointed to the cost-effectiveness of HPV testing in Brazil from the perspective of the Brazilian public health system.

### Recommendation

For women older than 30 years in Brazil, HPV testing alone should replace cytology. Cytology should be used as a triage test for cases of positive result on the HPV test = .

2. **Should cytology be offered at the same time as the HPV test in primary screening (co-testing)?**

When comparing the HPV test alone and combined with cytology (co-testing), the evidence shows that cytology does not substantially increase the detection of precursor lesions; therefore it does not provide extra reassurance. 11 13 23 25 26 27 28 29

### Recommendation

The HPV test alone is as effective as co-testing, and it has a lower cost. Co-testing is not recommended for primary screening.

3. **Should women between 25 and 29 years of age be screened by HPV testing?**

There is a consensus about the superiority of HPV testing in women older than 30 years of age. Many consider offering cytology alone for younger women due to the low specificity of the HPV test in women under the age of 30. Most of the positive results in young women reflect transient infections instead of precursor lesions. 7 30 Positive results in this group can lead to overdiagnosis. This issue can be solved by incorporating cytology as a triage test (after a positive HPV test and before colposcopy) or by genotyping HPV. 31 32

The critical issue is that, when offering cytology for women aged ≤ 30 years and HPV-testing for those older than that, both technologies would have to be available simultaneously, which would require more resources and induce their misuse. Thus, recent guidelines have updated the recommendation to start screening in women aged 25. 10 11 13 This strategy is reasonable in settings with limited resources and lack of a high-quality control process. 24

As for starting the screening before the age of 25 years, although the US Preventive Services Task Forces recommend starting at the age of 21, 15 there is evidence that before the age of 25 years, screening does not impact the prevention of cervical cancer. 14 31 The incidence and mortality rates of cervical cancer in women younger than 25 years are extremely low, and the high rate of transient infections and mild cytological abnormalities could lead to inadequate treatment. 30 32 33

### Recommendation

Women between 25 and 29 years of age should be screened by HPV testing. To reduce the risk of overdiagnosis, genotyping tests should be preferred in this situation. For those concerned about the effects on the outcomes of future pregnancies, a shared decision should be considered for the treatment when the risk of developing invasive lesions is low.

4. **Should cytology still be performed when an HPV test is positive? (HPV+ CYTO?)**

The HPV test is more sensitive than cytology, and should be used for primary screening. However, if screening were only based on HPV testing, patients would be refered to unnecessary colposcopies since most HPV infections are solved immunologically. Regarding cost-effectiveness, a second test before the colposcopy would improve the specificity. The recommendation is for a second test, or ‘triage test’, by cytology, preferably by liquid-based cytology using the same sample (reflex test). 6 9 29

In low- and middle-income countries, the reflex test can optimize the time and costs in screening, avoiding follow-up losses. Moreover, the reduction in unnecessary referrals to colposcopy is even more important in these countries.

### Recommendation

Cytology should be performed after a positive HPV test, to avoid unnecessary colposcopies. Liquid-cytology using the same sample (reflex test) is preferable, to avoid follow-up losses.

5. **What is the recommendation when the HPV test is positive and the cytology shows atypia? (HPV+ CYTO** **+** **: ?)**

The current recommendation is to perform colposcopy for all women. However, in women under the age of 30, for those with a previous negative HPV test and a new positive HPV test with triage cytology showing minor abnormalities (atypical squamous cells of undetermined significance – ASC-USs – or low squamous intraepithelial lesions – LSILs), it is possible to consider a more conservative management. 11 29 Follow-up in one year, rather than immediately referring to colposcopy, is recommended. This approach can be taken due to the low risk of precursor lesion. However, in settings where follow-up losses are high, immediate colposcopy should be considered.

### Recommendation

When the HPV test is positive and the cytology shows atypia, women should be referred to colposcopy. In women under 30 years of age, when follow-up is not a problem, a new HPV test in one year can be considered if the cytology shows ASC-US or LSIL.

6. **What is the recommendation when an HPV test is positive, the cytology shows atypia, but the colposcopy is normal? (HPV+ CYTO+ COLPO negative: ?)**

It is a consensus in the clinical practice that biopsies should assess any colposcopy changes. 8 11 Colposcopy impressions can be subjective. Studies 34 35 have shown a lack of intra- and inter-observer reliability, which reinforces the requirement of performing biopsies. When an HPV test is positive and the colposcopy is normal, the cytology result should guide the management.

When the colposcopy is normal and the cytology shows ASC-US or LSIL, there is no well-established recommendation yet. Cancer Council Australia 10 recommends a new HPV test in 12 months. This approach is possible because it is probably a transient infection rather than a real precursor lesion.

When the colposcopy is normal but the cytology result is atypical squamous cells, cannot exclude a high-grade lesion (ASC–H), atypical glandular cells (AGCs), or a highgrade squamous intraepithelial lesion or more severe (HSILþ), further assessment might be necessary. 10

Two additional procedures can improve diagnosis when the cytology is abnormal and the colposcopy is normal: Careful exclusion of vaginal intraepithelial neoplasia and the performance of an endocervical assessment by cytobrush or curettage, more importantly in type-3 transformation zone. 8 10 11 14

### Recommendation

There is no sufficient evidence to state a firm recommendation regarding discordant colposcopic impressions and other test results. Despite the lack of consensus, the cytology result can guide the management: if the cytology result shows ASC-US or LSIL, it is possible to repeat the HPV test in 12 months; if the cytology shows ASC-H or HSIL + , it is possible to refer the patient to an excisional procedure, waiting or not for the results of an endocervical assessment.

7. **It is possible to recommend an excision treatment bypassing a biopsy when the HPV test is positive, and cytology and colposcopy are abnormal? (HPV+ CYTO+ COLPO** **+** **: BIOPSY?)**

Biopsy results can guide the adequate management, to avoid overtreatment and detect lesions in early stages. For those cases in which the risk of HSIL is high, the colposcopy can be used without the biopsy confirmation to guide the excision of the transformation zone. 10 11 12 29 36

In the Brazilian setting, this approach is suitable due to the high estimation of follow-up losses. The 2016 INCA guidelines already stated that the biopsy can be skipped before the excision treatment when cytology and colposcopy are abnormal (screen-and-treat approach). 8 The HPV test provides more assurance regarding the risk of precursor lesion. In young women, when the specificity of the HPV test is lower due to the high prevalence of transient infection, the risk of overtreatment should be considered, and biopsy, recommended. 30 32

### Recommendation

In women older than 30 years of age, biopsy can be skipped before the excision treatment when the HPV test is positive and cytology and colposcopy are abnormal. In younger women, biopsy is recommended to avoid overtreatment.

8. **What should be the recommendation when the HPV test is positive, but the cytology is negative? (HPV+ CYTO negative: ?)**

When the HPVtest is positive and the cytology result is negative, the past tests should be accessed. If there is a history of previous positive or abnormal result, the recommendation is to refer to colposcopy due to the higher risk of persistent HPV infection. 10 29 If the past tests are not available or if they are all negative, follow-up in one year with HPV testing is recommended. 9 10 11

### Recommendation

When the HPV test is positive and the cytology is negative, follow-up on one year with a new HPV test is recommended. If there is previous history of positive HPV test or cytology, the women should be referred to colposcopy even when the cytology is negative.

9. **What is the recommendation when an HPV genotyping test is positive for types 16 or 18? (HPV 16/18** **+** **: ?)**

Although genotyping is not crucial, it is well defined that HPV subtypes 16 and 18 present the highest risks for the development of high-grade lesions or occult cancers. 30 When genotyping is available and the result is positive for types 16 or 18, the recommendation is to refer to colposcopy, regardless the result of the cytology test. 9 11 If the cytology is not performed as a triage test, it can be collected during the colposcopy visit.

### Recommendation

When an HPV genotyping test is positive for types 16 or 18, the women should be immediately referred to colposcopy, regardless the result of the cytology test. If the colposcopy shows mild abnormalities, a biopsy should be performed. If the colposcopy suggests a high-grade or a more severe lesion, the excision treatment is preferable, except in women under 25 years of age, when a biopsy is mandatory.

10. **What is the recommendation when the HPV genotyping test is positive for types 16 or 18, but the colposcopy is normal? (HPV16/18** **+** **, Colpo negative: ?)**

The cytology result should be confirmed in this situation, even if by a reflex test or by cytology collected during the colposcopy visit. The risk of HSIL+ is high for positive results for types 16 or 18, so treatment can be expedited if the cytology shows ASC-H or HSIL + , even when the result of the bipsy is not available. 10 11 29 37 When the cytology result is negative, follow-up should include a new cytology and colposcopy examinations in six months and a new-HPV test in one year.

### Recommendation

When the HPV test is positive for types 16 or 18 and the colposcopy is normal, the result of the cytology test should be confirmed to guide the subsequent approach. If the cytology result is negative (ASC-US or LSIL), follow-up in one year with an HPV test is recommended. If the cytology result shows HSIL + , excision should be considered. In case of a negative cytology result, follow-up should include new cytology and colposcopy examinations in six months and a new HPV test in one year.

11. **When to stop screening when HPV testing is available?**

An observational cohort study 38 has shown that, in women aged 50 years or older with previous negative HPV test results, the probability of detecting cervical cancer on screening is low. Thus, the continuation of screening may be cost-ineffective. So, if there is a series of negative test results and adequate surveillance spanning 10 years in women older then 65 years of age, the screening should be discontinued. 8 9 11 15 The risk-estimate approach suggests that if there is history of HSIL + , the risk persists until at least 25 years, 29 so it is reasonable to keep screening until life expectancy.

### Recommendation

Screening should be discontinued in patients older than 65 years of age if there has been adequate surveillance for the past 10 years. If there is a history of HSIL + , screening should be continued until the life expectancy or at least 25 years of the treatment.

12. **How should we perform screening in pregnant women?**

The risk of precursor lesions does not increase during pregnancy, neither do the rates of progression to cancer. Therefore, screening, either by an HPV test or by cytology, should follow the regular surveillance. 8 9 10 11 14 It is important to note that the colposcopy impression could be affected due to physiologic pregnancy changes. A diagnostic excisional procedure or biopsy is only accepted if cancer is suspected. 8 10 11 14 When abnormalities are present, there is a suggestion to evaluate the cervix every 3 months during pregnancy, and to perform final assessment at about 90 days after delivery. 8 10

### Recommendation

Pregnant women should be managed as non-pregnant women regarding screening. Biopsies are only accepted if cancer is suspected.

13. **How should we perform the screening in immunocompromised women?**

There is sufficient evidence that immunocompromised women have higher risks of developing precursor lesions. 39 40 41 Conservative approaches should be an option only when the immunological status is satisfactory and stable, and close follow-up is warranted.

### Recommendation

The screening in immunocompromised women should start within 1 year of the first sexual intercourse, each 6 to 12 months, with cytology for women under the age of 25 and HPV tests in women older than 25 years. Management should consider that this group has a high risk of developing HSIL + .

14. **How should we perform the screening in vaccinated women?**

According to the proportion of uptake among the population, vaccination against oncogenic HPV genotypes can significantly decrease the rates of precursor lesions and cervical cancer. 42 43 However, the coverage of vaccination programs is heterogeneous and fluctuates according to social, economic, and ethnic aspects, and has not yet reached the target levels, especially in low- and middle-income countries. 44 45 46 47

In Brazil, vaccines have been available in private clinics since 2006, but were only incorporated in the Brazilian Unified Health Care System in 2014. As soon as vaccination coverage increases, the expectations are to combine the screening and vaccination strategies. Some countries have recently updated their guidelines, suggesting that the vaccinated population can start the screening later. 14 42 45 However, considering the variations in coverage, other guidelines still do not recommend changing the screening in the vaccinated population. 11 12 15

### Recommendation

The screening in vaccinated women should be the same as in non-vaccinated women, since the coverage rates are still low and have not reached safe levels among the screened populations to suggest changes on the recommendations.
